# Retinoic Acid: The Autacoid for All Seasons

**DOI:** 10.3390/nu14214526

**Published:** 2022-10-27

**Authors:** Joseph L. Napoli

**Affiliations:** Graduate Program in Metabolic Biology, Department of Nutritional Sciences and Toxicology, The University of California-Berkeley, Berkeley, CA 94704, USA; jna@berkeley.edu

All-trans-retinoic acid (RA), a metabolite of vitamin A (retinol), exerts profuse actions that enable multiple aspects of reproduction, embryonic development and post-natal regulation of energy metabolism, glucoregulatory control, organ function, and of the skeletal, immune, nervous and cardiovascular systems, as well as cell proliferation vs. differentiation. RA promotes or prevents differentiation of stem cells, and regulates functions of differentiated cells. RA achieves these extensive and sometimes conflicting actions through activating up to four nuclear receptors (RARα, β, γ and PPARβ/δ), and also through non-genomic mechanisms, which involve RA-specific cellular binding-proteins (CRBP1, CRABP1 and 2, FABP5) [1]. A cell’s response to RA relies on its specific expression of nuclear receptors, binding proteins and homeostatic enzymes. Such expression, based on these complex permutations, guides both genomic and non-genomic actions, and allows different cell types to respond uniquely to RA signaling. RA homeostasis derives from actions of cellular retinol-binding proteins (CRBP1,2), multiple retinol and retinal dehydrogenases (RDH1/16, RDH10, DHRS9 and RALDH1, 2, 3), retinal reductases (DHRS3, DHRS4, RDH11), retinol esterification enzymes (LRAT, DGAT1), retinyl ester hydrolases (REH), and RA catabolic enzymes (CYP26A1, B1, C1). In addition, specific plasma membrane receptors (SRA6, RBPR2) facilitate access of retinol to cells, whereas the carotene receptor (SR-B1) facilitates cell uptake of pre-vitamin A carotenoids. Another tier of regulation involves positive feedback by RA to induce LRAT and CYP26, and inhibition of LRAT by apo-CRBP1. Consequently, the RA concentration controls the disposition of retinol, whether directed primarily to esters vs. RA biosynthesis. RA catabolism reacts sensitively to RA concentrations to add another rheostat. Hormesis also rules RA actions, i.e., RA concentrations affect the nature of its behavior. Physiological, pharmacological and toxic effects can be distinct; moreover, some effects of vitamin A-deficiency can mimic effects of RA toxicity. Androgens and estrogens interact with RA. Estrogen promotes RA potency, whereas RA retards estrogen potency. RA induces androgen biosynthesis, but androgens affect RA receptors negatively. The RA isomer, 9-cis-RA, and perhaps other naturally occurring retinoids, contribute to select retinoid actions. This complexity of the RA metabolon and its interactions with steroid hormones reveals how RA exerts paradoxical actions on distinct cell types and exerts sexual dimorphism.

Vertebrate life cannot exist without RA. RA conceivably represents the signaling effector in all vertebrates with the most extensive activities. RA targets rely unquestionably on a blend of more receptors, more complex homeostasis, and further sites of biosynthesis and signaling than other effectors. Interest in RA, however, remains relatively modest among biologists. Perhaps, this arises from a combination of unfamiliarity with non-visual vitamin A function, retinoid toxicity, and hype generated regarding cancer chemoprevention aspects of retinoids. Decades ago, the ability of RA and its analogs (synthetic retinoids) to prevent carcinogenesis in vitro and to reverse tumorigenicity of cultured cells encouraged expectations that retinoids would provide wonder drugs to reduce, if not to eradicate, the scourge of cancer. This enthusiasm did not regard sufficiently the complexity of RA homeostasis and actions, retinoid toxicity and pharmacokinetics. Hype ultimately engenders disappointment. Disappointment ultimately engenders excessively negative counter-assessment. Retinoids never reached their unrealistically presumed potential for preventing and treating cancer (but do contribute to cancer treatment). This, along with toxicity and costs of developing therapeutics for cancer and other applications chilled a “hot” area to an underrated specialty. Nevertheless, as research continues to expose RA targets and uses of synthetic retinoids, this field seems on the cusp of major expansion [2].

Articles in this Special Issue of *Nutrients* entitled “Retinoids and Human Health–Current Roles and Future Directions” relate many biological functions of retinoids. Not all areas of retinoid action could be covered. Harrison details actions of BCO1 and BCO2 in converting carotenoids into retinoids and apo-carotenals, respectively, and the retinoid-like metabolism of apo-carotenals. Notably, β-apo-20,14’-carotenoids and β-apo-13-carotenone bind to purified RAR with high affinity and antagonize RA activity in transactivation assays and target genes. Although the carotenals and their metabolites occur extensively in vivo, their pathophysiologic relevance to human health has not been established. This essay reveals an area of potential significance, which may be realized as technological development and biological insight advance. The articles by Napoli and by O’Connor et al. present complementing perspectives of retinoid metabolites, the retinoid metabolon and retinoid mechanisms of action, and their effects on retinoid function and human health. The former includes sexually dimorphic actions of RA and its pro-beta cell, but anti-insulin effects, and its interactions with sex hormones. The latter includes details of vitamin A absorption and distribution. The article by Yabut and Isoherranen focuses on delivery of RA by CRABP1 and CRABP2 to CYP26 catabolic enzymes. These latter three articles provide broad insight into RA homeostasis, its regulation, and its diverse and complex actions. These articles provide background for the report by Czuba et al. about retinoid concentrations in blood during stages of pregnancy.

The essay by Nhieu et al. discusses relevance of CRABP1 to human health by expanding beyond its delivery of RA to catabolic enzymes, to mediation of non-canonical mechanisms. The CRABP1 signalosome suppresses tumorigenesis by dampening MAPK kinase signaling, which represses the cell cycle to reduce proliferation of neural and embryonic stem cells and ameliorate inflammation. A CRABP1 signalosome also regulates CaMKII in cardiomyocytes and motor neurons. Inhibition of ERK activity by CRABP1 inhibits adipogenesis and adipose hypertrophy. Crabp1 knock-out mice fed a high-fat diet experience increased obesity and insulin resistance, relative to wild type.

Melis et al. focus on the relationship of liver retinoids, retinoid metabolism, and RAR signaling to liver health and disease prevention, such as hepatocellular carcinoma, steatohepatitis, and non-alcohol and alcohol-associated fatty liver diseases. This article discusses evidence that 4-oxo-RA and 4-OH-RA serve as essential retinoids at specific sites, such as maintaining hematopoietic stem cells; and extends the discussion of two anti-insulin actions of RA—retarding de novo lipogenesis and promoting lipid oxidation.

Steinhoff et al. takes us up to date regarding the lipocalin RBP4, the serum retinol-binding protein. Although many tissues produce RBP4, liver hepatocytes seem the sole source of the RBP4-retinol complex secreted into serum. The visual cycle relies on the serum complex for optimal delivery of retinol to the retina. Mutations of RBP4 in humans link to impaired vision. The report by Radhakrishnan et al. extends this insight by revealing that ablation of RBPR2 causes loss of vision.

Steinhoff et al. also report that higher concentrations of the serum RBP4-retinol complex impair insulin signaling in muscle and induce phosphorylation of STRA6 in adipose, activating JAK2, which activates STAT5. This cascade impairs insulin action in adipose, and may affect muscle. Distinctly, RBP4 expressed within extrahepatic tissues seems to function as an intracrine agent. For example, adipose RBP4 provokes inflammation within adipose tissue to impair insulin sensitivity.

Sidell and Kane concentrate on RA’s immunostimulatory properties by discussing intestinal mucosal dendritic cells as sources of RA. They relate that HIV/SIV infection links closely to disruption of RA-regulated gut immune cells. Decreases in gut mucosal RA permit HIV/SIV infection. Anti-retroviral therapy does not reverse completely the decrease in RA biosynthesis that contributes to immune cell dysfunction. The authors suggest therapeutic use of retinoids to counteract HIV-associated intestinal disease as a component of a “shock and kill” strategy to counter viral infection. The report by Chai et al. implicates reduced vitamin A status with compromised immune function associated with increased mortality.

Schleif et al. review effects of RA on male and female gamete formation and their regulation. RA programs formation of the blood-testis barrier, spermatogonial differentiation, and spermiation, and assists in meiotic completion. Because spermatogenesis requires RA, inhibition of RA biosynthesis or antagonism of function may provide an approach to male contraception. Dosing the aldehyde dehydrogenase inhibitor bis-(dichloroacetyl)-diamine, for example, prevents spermatogenesis, but also impairs alcohol metabolism. Ablating key RA-dependent genes causes irreversible sterility in mice, but a pan-RA receptor antagonist induces reversible sterility. These studies establish “proof of principle”, but also reveal need for more targeted strategies. In contrast to fundamental insight into spermatogenesis, research into oogenesis has spawned conflicting results. The prevailing evidence, however, confirms the reliance of the human female reproductive system on RA for development and health.

VanBuren and Everts direct attention to RA effects on skin and hair. RA exerts dose- and time-dependent effects over hair follicle and melanocyte stem cells, shapes the hair cycle and promotes wound healing. RA effects on hair follicle stem cells seems to pursue a U-shaped curve, presenting a classic example of hormesis. Interestingly, skin also relies on retinal, which had been associated only with vision.

Epidermal melanocytes and keratinocytes express four opsins, which require retinal to function. Opsin-retinal conjugates induce UV-stimulated melanin synthesis in a manner mechanistically similar to their actions in the visual cycle.

Altogether these articles present the complexity of the retinoid metabolon and mechanisms of retinoid action. Fascinating points include the sexual dimorphism of RA, and data that retinoid binding-proteins serve beyond chaperoning retinoid metabolism, but also exert non-genomic behaviors essential to retinoid function. Possible contributions of apo-carotenals to retinoid function also offer intriguing insight, as well as potential contributions of endogenous RA metabolites to retinoid function, and occurrence of opsin-retinal conjugates in skin. RA’s anti-viral and liver health-promoting activities suggests uses not imagined previously. Obstacles to widespread retinoid therapy remain, but progress continues on many fronts. Application of life-preserving retinoid therapy remains in its infancy, but promises new therapeutics, and at least, a grasp of the essential contributions of retinoids to human health.
